# Enhancing groundwater quality assessment in coastal area: A hybrid modeling approach

**DOI:** 10.1016/j.heliyon.2024.e33082

**Published:** 2024-06-19

**Authors:** Md Galal Uddin, M.M. Shah Porun Rana, Mir Talas Mahammad Diganta, Apoorva Bamal, Abdul Majed Sajib, Mohamed Abioui, Molla Rahman Shaibur, S.M. Ashekuzzaman, Mohammad Reza Nikoo, Azizur Rahman, Md Moniruzzaman, Agnieszka I. Olbert

**Affiliations:** a School of Engineering, University of Galway, Ireland; b Ryan Institute, University of Galway, Ireland; c MaREI Research Centre, University of Galway, Ireland; d Eco-HydroInformatics Research Group (EHIRG), Civil Engineering, University of Galway, Ireland; e The Department of Geography and Environment, Jagannath University, Dhaka, Bangladesh; f Geosciences, Environment and Geomatics Laboratory (GEG), Department of Earth Sciences, Faculty of Sciences, Ibnou Zohr University, Agadir, Morocco; g MARE-Marine and Environmental Sciences Centre-Sedimentary Geology Group, Department of Earth Sciences, Faculty of Sciences and Technology, University of Coimbra, Coimbra, Portugal; h Laboratory for Sustainable Innovation and Applied Research, Universiapolis—International University of Agadir, Agadir, Morocco; i Laboratory of Environmental Chemistry, Department of Environmental Science and Technology, Faculty of Applied Science and Technology, Jashore University of Science and Technology, Jashore, 7408, Bangladesh; j Department of Civil, Structural and Environmental Engineering, and Sustainable Infrastructure Research & Innovation Group, Munster Technological University, Cork, Ireland; k Department of Civil and Architectural Engineering, Sultan Qaboos University, Muscat, Oman; l School of Computing, Mathematics and Engineering, Charles Sturt University, Wagga Wagga, Australia; m The Gulbali Institute of Agriculture, Water and Environment, Charles Sturt University, Wagga Wagga, Australia

**Keywords:** Groundwater, RMS-WQI model, Machine learning, Water quality index (WQI), Uncertainty

## Abstract

Monitoring of groundwater (GW) resources in coastal areas is vital for human needs, agriculture, ecosystems, securing water supply, biodiversity, and environmental sustainability. Although the utilization of water quality index (WQI) models has proven effective in monitoring GW resources, it has faced substantial criticism due to its inconsistent outcomes, prompting the need for more reliable assessment methods. Therefore, this study addressed this concern by employing the data-driven root mean squared (RMS) models to evaluate groundwater quality (GWQ) in the coastal Bhola district near the Bay of Bengal, Bangladesh. To enhance the reliability of the RMS-WQI model, the research incorporated the extreme gradient boosting (XGBoost) machine learning (ML) algorithm. For the assessment of GWQ, the study utilized eleven crucial indicators, including turbidity (TURB), electric conductivity (EC), pH, total dissolved solids (TDS), nitrate (NO_3_^−^), ammonium (NH_4_^+^), sodium (Na), potassium (K), magnesium (Mg), calcium (Ca), and iron (Fe). In terms of the GW indicators, concentration of K, Ca and Mg exceeded the guideline limit in the collected GW samples. The computed RMS-WQI scores ranged from 54.3 to 72.1, with an average of 65.2, categorizing all sampling sites' GWQ as “fair.” In terms of model reliability, XGBoost demonstrated exceptional sensitivity (R^2^ = 0.97) in predicting GWQ accurately. Furthermore, the RMS-WQI model exhibited minimal uncertainty (<1 %) in predicting WQI scores. These findings implied the efficacy of the RMS-WQI model in accurately assessing GWQ in coastal areas, that would ultimately assist regional environmental managers and strategic planners for effective monitoring and sustainable management of coastal GW resources.

## 1 Introduction

Groundwater (GW) is an invaluable natural resource stored in the critical zone of the Earth's asthenosphere [1]. It plays a fundamental role in the hydrological cycle, biodiversity, and ecology. Roughly one-third of the world's fresh water supply originates from GW, with domestic, agricultural, and industrial sectors utilizing 36 %, 42 %, and 27 % of it, respectively [2]. However, the quality of GW is under significant threat due to an intricate combination of human activities and climatic factors especially in coastal areas, as highlighted in recent studies [[3], [4], [5], [6], [7]]. Various pressures contribute to GW pollution, including industrial discharge, agricultural runoff, improper waste disposal, and urban development [8,9]. Industrial activities release harmful chemicals and pollutants into the ground, contaminating the GW supply [[10], [11], [12]]. Similarly, agricultural practices involving excessive use of fertilizers, pesticides, and herbicides result in the seepage of harmful substances into the GW [[13], [14], [15]]. Improper waste disposal methods, especially in densely populated urban areas, lead to the infiltration of pollutants into the soil, eventually reaching the groundwater reservoirs [[16], [17], [18], [19]]. In addition to that, climate change exacerbates these issues by altering precipitation patterns, causing shifts in GW recharge areas, and promoting sea-level rise, leading to saline intrusion in coastal regions [20,21]. Increased temperature can also intensify the chemical reactions in the soil, further affecting GWQ [22]. Therefore, addressing these challenges requires a holistic approach to manage GW resources sustainably.

Being an issue of global significance, monitoring of GW state has been highlighted in the United Nation's (UN) Sustainable Development Goal (SDG) no. 6 (ensure availability and sustainable management of water and sanitation for all by 2030). Following UN's SDG approach, most of the developed and developing countries in the globe employed the manual procedures to establish the water quality (WQ) states in comparison between measured/field/lab test concentrations and guidelines values of WQ indicators [[23], [24], [25], [26]]. Nevertheless, several recent studies have indicated that the manual technique may have significantly increased the level of uncertainty in the final evaluation of the water state [[27], [28], [29], [30], [31], [32], [33]]. To resolve this issue, a variety of instruments and models have been developed to this date to evaluate and determine the GW state. Among these, the WQI model is one of the widely utilized tools [[34], [35], [36]]. Typically, WQI models utilize mathematical procedures to convert available WQ indicator monitoring data into a single value that is expressed to utilize as an indicator of WQ. Architecturally, WQI models include five components: (i) indicator selection, (ii) indicator sub-indexing, (iii) weighting, (iv) index aggregation and (v) classification scheme [34]. The sub-index function, which enables the transformation of indicator concentrations into dimensionless values, is another crucial element of a WQI model. Both experts and novices may easily understand the WQI's outcomes because of its straight-forward mathematical architecture. To this date, several WQI models have been developed by numerous nations, organizations, and researchers to evaluate the WQ for a variety of uses, including irrigation, GW, and surface waters (lakes, rivers, etc.) [34,35]. Recent research from multiple investigations has shown that the existing WQI models have demonstrated a significant level of uncertainty due to their own structures [34,[37], [38], [39], [40]]. Moreover, the eclipsing, ambiguity and metaphoring issues generated substantial bias in the WQI's final output. Most recently, authors [41] developed an unweighted data-driven RMS-WQI model and a weighted data-driven approach Irish Water Quality Index (IEWQI) model [39], whereas these models have proven effective in reducing issues related to model eclipsing, ambiguity, metaphoring issues and data outliers [42]. Furthermore, the RMS-WQI model demonstrated superiority in rating WQ of both surface water [43] and GW resources [38].

In recent years, the fusion of Machine Learning (ML) and Artificial Intelligence (AI) has become integral in addressing complex environmental challenges, especially in the intricate task of predicting GWQ [38,[44], [45], [46]]. A diverse array of algorithms, such as Random Forest (RF), Decision Trees (DT), k-Nearest Neighbors (KNN), Support Vector Machines/Regression (SVM/SVR), XGBoost, and Gaussian Processes Regression (GPR), constitutes a versatile toolkit for analysts to navigate intricate GW datasets and elevate forecasting precision. The RF utilizes an ensemble approach, excels in capturing intricate non-linear relationships, while DT offers interpretability, shedding light on decision-making processes despite a susceptibility to overfitting. On the other hand, KNN relies on data point proximity, proves adept at discerning localized patterns in GWQ variations. The SVM/SVR with their high-dimensional adaptability, demonstrated robust performance in handling complex relationships. On the contrary, XGBoost stands out for its ability to sequentially refine model predictions, often yielding superior accuracy [47] whereas GPR excels in capturing uncertainty and provides a probabilistic framework for understanding GWQ variations [6]. Nevertheless, each of the above-mentioned algorithms present distinctive challenges. To give an example, RF may pose interpretability challenges due to their ensemble nature while DT may grapple with overfitting concerns [48]. KNN's efficacy hinges on careful parameter selection and GPR may encounter computational challenges with large datasets [49,50]. The SVM/SVR may exhibit sensitivity to kernel choice and requires meticulous tuning [51,52]. Although XGBoost is substantially robust, it demands careful consideration of hyperparameters. However, recent research has emphasized the utility of the XGBoost model in predicting WQ, highlighting its effectiveness in the context of GWQ prediction [26,32,33,39,53]. Therefore, considering the above discussion, this study leveraged the XGBoost algorithm to predict RMS-WQI scores, evaluating the model's sensitivity in interpreting WQI model inputs into scores. This underscores the importance of informed model selection, acknowledging both the strengths and limitations of these algorithms in enhancing our understanding and management of GWQ.

In terms of a developing country like Bangladesh, it is worth mentioning that nearly 97 % of its population relies on GW resources for meeting the demands of drinking water [11]. The coastal region of Bangladesh is remarkably vulnerable to several catastrophes, and the effects of climate change are affecting new aspects of life and agriculture [[54], [55], [56]]. Elevated levels of salt content in the drinking water have an impact on about 20 million coastal residents of Bangladesh [[57], [58]]. Besides, Bangladesh experience altered rainfall patterns and increased flood frequency that causes saltwater intrusion in coastal regions, which consequently contaminate GW resources [59]. Numerous studies have recently confirmed the degradation of WQ in various coastal regions of Bangladesh, including Satkhira [60], Khulna [61,62], Patuakhali [63], Gopalganj [64,65] and Jashore [66]. However, being the only island district of Bangladesh, the GW resources of Bhola have gained lesser attention of the scientific community in terms of rating GW state. Although a few studies have been conducted to assess the GWQ of Bhola, none of these studies have utilized the state-of-the-art WQI modeling approaches [4,67]. Therefore, the present study utilized the RMS-WQI model for assessing the GWQ of Bhola. Additionally, to the best of the authors knowledge, the research is the maiden initiative to assess coastal GWQ using recently innovated RMS-WQI approaches incorporating ML/AI techniques. Therefore, the research aim was to evaluate the coastal GWQ of Bhola incorporating RMS-WQI model. The aim of this study achieved through the following objectives:

• To compute the WQI scores using advanced RMS-WQI approaches.
• To evaluate the model performance utilizing the ML/AI approaches in terms of model uncertainty and sensitivity.
• To rate GWQ using a new classification scheme in terms of drinking suitability in order to reliable assessment.

The implications of this research extend beyond its immediate findings. The integration of ML/AI techniques with RMS-WQI approaches not only enhances the accuracy and reliability of GWQ assessments but also sets a precedent for future research endeavors in the refinement of the RMS-WQI model. As the research pioneers the intersection of RMS-WQI, ML/AI, and GWQ assessment, it lays the foundation for continued advancements in the monitoring and management of water resources, offering valuable insights for both researchers and practitioners.

## 2 Materials and methods

### 2.1 Description of the study area

**Fig. 1:**
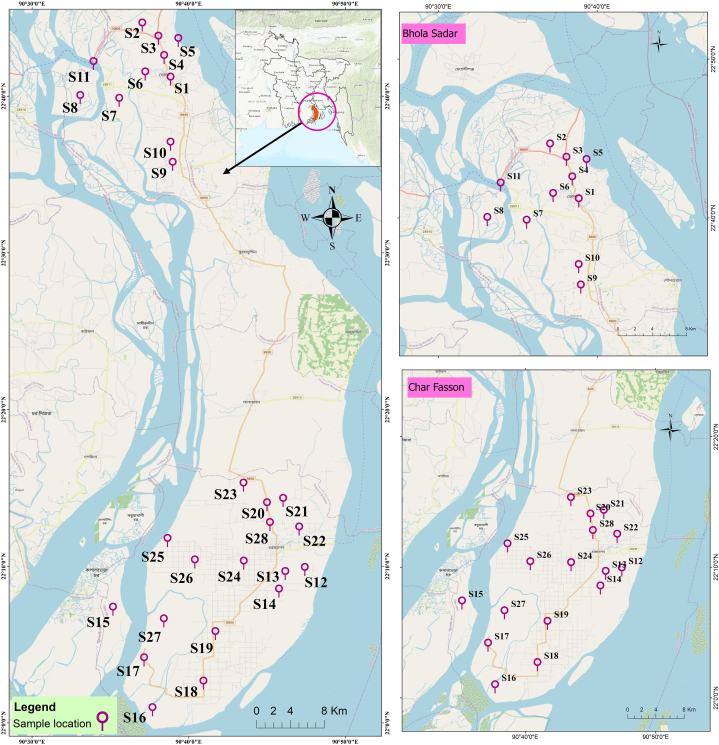
Study domain and sampling sites in Bhola district.

Bhola, the largest island in Bangladesh, is situated along the coastal region and is predominantly surrounded by water features (Fig. 1). Covering an area of approximately 3737.21 square kilometers in the southern part of the Barisal division, Bhola comprises 5 municipalities, 9 thanas, 66 unions, and 473 villages. With an agricultural production rate of about 63.64 % and a literacy rate of 47 %, Bhola exhibits a tropical wet and dry or savanna climate (Aw classification) at an elevation of 0 m (0 feet) above sea level. The average annual temperature ranges from 12 to 32.8 °C (Fig. S1). Bhola experiences an annual precipitation of around 191.45 mm, peaking in June (465.2 mm) and reaching its lowest in January (10.3 mm) (Fig. S1). Geographically, Bhola is surrounded by the Patuakhali district and the Tetulia river to the west, the Lakshmipur and Noakhali districts, the lower Meghna River, and the Shahbazpur channel to the east, the Bay of Bengal to the south, and the Lakshmipur and Barisal districts to the north [68]. The groundwater level in the region fluctuates between 850 and 1400 feet below the surface. Despite its coastal nature, Bhola has a significant agricultural output, and GW from the Deep Tubewell (DTW) is considered predominantly fresh for drinking and irrigation purposes [55]. This intriguing characteristic motivated the selection of Bhola as the research region for our GW analysis.

### 2.2 Data description

The study relied on WQ data sourced from the investigation conducted by Ref. [4]. The authors of Ref [4] succinctly outlined the procedures for collecting GW samples and analyzing WQ indicators, encompassing TURB, EC, pH, TDS, NO_3_^−^, NH_4_^+^, Na, K, Ca, Mg, and Fe. These indicators were obtained from GW samples collected from twenty-eight sampling sites, among which eleven sampling sites were from Bhola Sadar and the remaining seventeen sites were in Char Fasson upazila which is the biggest and most populated sub-district under Bhola district. For detailed of the selection of the sampling sites, sample collection and analytical procedure, please refer to Ref. [4]. Fig. 1 and Table S1 depict the geographical distribution of the sampling sites within the study domain. For a comprehensive overview, Table S2 presents information on various WQ indicators, their units, and corresponding guideline values.

### 2.3 Computing RMS-WQI scores

A range of methods are employed in the literature to compute WQI scores, and the WQI model stands out as one of the most widely used approaches. The WQI model facilitates the conversion of multiple WQ indicators into a unit-less numerical value, known as the “WQI score” allowing for the assessment of WQ for purposes such as irrigation, GWQ, and river WQ. These models are typically categorized into two groups: (i) weighted and (ii) unweighted. Unlike weighted models, unweighted models consist of four components: (1) indicator selection method, (2) sub-index functions, (3) aggregation function, and (4) classification schemes. Recent studies have revealed significant uncertainty in assessment results due to the model architecture, with details on various limitations found in Ref. [34].

**Fig. 2:**
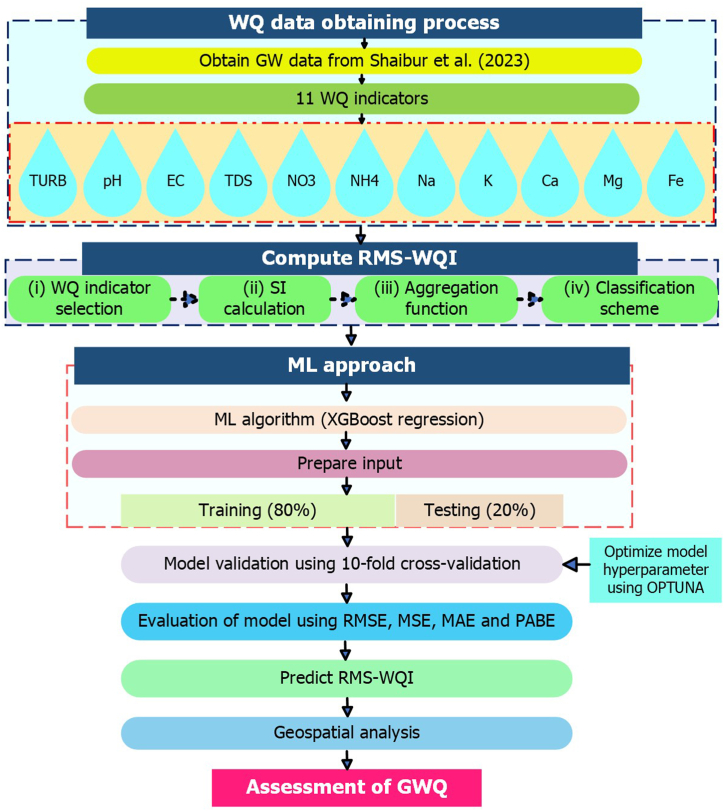
A comprehensive framework for adopting improved WQI approaches for the assessment of GW.

Recently, authors have conducted a study comparing weighted and unweighted WQI approaches for assessing coastal waters [47]. This study indicates no considerable differences between the two approaches and reports that the unweighted RMS-WQI approach is free from model ambiguity and eclipsing issues and thus, ensures a more accurate rating of WQ. Additionally, recent research suggests that the weighting component contributes significant uncertainty to outcomes, emphasizing the effectiveness of unweighted approaches for more accurate WQI scores [47]. Therefore, the study utilizes the novel unweighted RMS-WQI model for computing WQI scores, thoroughly explained in Fig. 2. The RMS-WQI model can be expressed as follows:

(1) $$\text{RMS}\;–\;\text{WQI}=\sqrt{\frac{1}{n}\sum_{i=1}^{n}s_{i}^{2}}$$

Here *S*_*i*_ is the sub-index of *i*^*th*^ WQ indicators, and *n* refers to the number of WQ indicator. After computing the WQI scores, they are translated into the rank of WQ using Table 1.

**Table 1:** Classification categories of the RMS-WQI model for GW according to Ref. [38].

GW quality	GWQ score range	Explanation
Good	80–100	All WQ indicators meet standards, and water is suitable for all purposes. WQ may have no impact on human health.
Fair	50–79	Few WQ indicators breach the standards. WQ is moderately vulnerable for human health, however, long-term use of this kind of water may create different health issues; therefore, water must be purified before use to avoid health risks.
Marginal	30–49	Majority of WQ indicators breach standards and must be used with precaution before being using for any sort of purpose. WQ is highly vulnerable to human health.
Poor	0–29	All WQ indicators breach the standard, and water is not suitable for any purpose. WQ is severely vulnerable to human health.

#### 2.3.1 RMS-WQI score translation scheme

The ultimate objective of the WQI approach is to assess the WQ based on the computed WQI score(s). According to literature, several classification schemes are available for determining the WQ state [42]. However, significant anomalies have been reported by several studies due to inappropriate classification schemes. To address this issue, Ref. [33] have identified the metaphoring issue within the existing classification scheme(s) and introduced a novel classification scheme for surface water which was later modified for rating GW quality [38]. To this date, a number of studies have confirmed the effectiveness of this approach for assessing the GW quality [38]. Therefore, the present study followed the classification scheme of Ref. [38] for translating the RMS-WQI score (Table 1).

### 2.4 Model sensitivity analysis -prediction approach

#### 2.4.1 Data pre-processes

Data standardization plays a pivotal role in ML/AI model development by averting issues of model overfitting or underfitting, thereby ensuring optimal performance [24,32,41,69,70]. Standardized data facilitates uniform comparison and interpretation of features, expediting the convergence of ML/AI algorithms during training. Common techniques like Z-score normalization, Min-Max scaling, and Robust scaling effectively adapt input features to a consistent scale. The choice of standardization method depends on the dataset's specific characteristics and the ML/AI model requirements. In the context of WQ assessment, the research utilized the normalization method developed by Ref. [24] to standardize WQ indicators before applying ML and AI models, highlighting the critical importance of this preprocessing step for enhanced efficiency and accuracy.

#### 2.4.2 Input preparation

The method described in Ref. [32] was used to create 10,000 random samples before creating the ML models. Ref. [32] provides further information on the processes. Following the steps outlined in Ref. [32], we split the WQ datasets into two subsets: 80 % (8000 samples) for training and 20 % (2000 samples) for testing to create the prediction models. The ML models were successfully created, and we then utilized them to forecast the WQI scores for the twenty-eight sample locations.

#### 2.4.3 ML model development

In this study, the prediction of RMS-WQI scores was conducted using the XGBoost ML algorithm. The selection of XGBoost was informed by the authors' prior research [6,24,33,36,39,41,47] and supported by findings from other investigations [[71], [72], [73], [74]] that demonstrated its superior performance. The implementation of the XGBoost model followed the approaches outlined in Ref. [24], with the optuna (OPT) strategy employed for hyper-parameter tuning. Optuna, a widely used technique in machine learning research [36,39,[75], [76], [77]], was utilized to maximize the model's performance. For further details on the XGBoost model and optimization process, refer to Ref. [24]. All ML and statistical analyses, along with associated figures, were conducted using the Python programming language on the Google Colab cloud-computing platform. This environment proves highly efficient for implementing diverse cutting-edge ML models and statistical analyses in Python.

#### 2.4.4 Model performance analysis

The cross-validation strategy is widely employed in the literature to assess the performance of ML models [24,32,41,[75], [76], [77]]. Following Ref. [24]'s methodology, the current study utilized the 10-fold cross-validation procedure, with details provided by Ref. [24]. Performance assessment in this study involved four commonly used measures: root mean squared error (RMSE), mean absolute error (MAE), mean square error (MSE), and Percentage of Absolute Bias Error (PABE). Ref. [35] offers comprehensive details on these performance measures and their notations. Smaller values closer to zero are generally recommended for enhanced model performance [25,39,41,78].

#### 2.4.5 Model uncertainty analysis

Several studies have employed Monte Carlo Simulation (MCS), a widely recognized method for modeling uncertainty in computational models [32,[79], [80], [81], [82]]. Following a unique methodology proposed by Ref. [32] for measuring WQI model uncertainty using MCS and ML techniques, this research marked the first systematic attempt to estimate uncertainty in WQI approaches. The research utilized Ref. [32] approaches for estimating the model uncertainty. The protocol details of the methodology can be found in Ref. [32]. In addition, to enhance model performance, 10,000 random samples were generated using MCS techniques before conducting the uncertainty analysis. The findings of the RMS-WQI models' uncertainty, illustrated in Fig. 7, were presented using probability density function (PDF) and cumulative density function (CDF).

#### 2.4.6 Evaluation model efficiency

Numerous metrics that are often used in literature, such as the Nash-Sutcliffe efficiency (NSE) and the model efficiency factor (MEF), are used to assess the model's effectiveness [83,84]. These metrics have been used in a number of recent WQ studies to evaluate the efficacy of the WQ model [36,39,83,85,86]. According to Ref. [36], the NSE and MEF were employed in this work to assess the model's effectiveness regarding the spatial resolution of the study domain. In Ref. [36], the methods for utilizing NSE and MEF are further described. In terms of interpretation, a smaller MEF score denotes a better (bias-free) model, whereas an NSE value of 1 often denotes an “excellent” model [36,83,84].

#### 2.4.7 Model eclipsing and ambiguity analysis

Recently several studies highlighted the inconsistent results in WQI models attributed to issues of model eclipsing and ambiguity. Ref. [34] delve into the specifics of these challenges in WQI approaches. Recently, the authors introduced a pioneering methodology for assessing model eclipsing and ambiguity, particularly focused on transitional and coastal waters. This innovative approach represents a comprehensive effort, marking the first systematic attempt to understand and address model eclipsing and ambiguity. In this study, Ref. [41] methodology was employed to evaluate the model's challenges related to eclipsing and ambiguity, with detailed insights available in Ref. [41].

### 2.5 Geospatial assessment of GW

A geospatial analysis also has been adopted in this research to know the spatial variation of WQI score within the studydomain [11]. Kriging, a geostatistical interpolation technique, is a potent tool for estimating values at unseen places by taking into account the spatial autocorrelation of a variable. For the purposes of evaluating the GWQ in terms of spatial resolution, present study utilized the Empirical Bayesian Kriging (EBK) following the approach of Ref. [35] through the most recent geographic information systems (GIS) program (ArcGIS 10.5 software).

## 3 Results and discussion

### 3.1 Statistical attributes of GWQ indicators

**Fig. 3:**
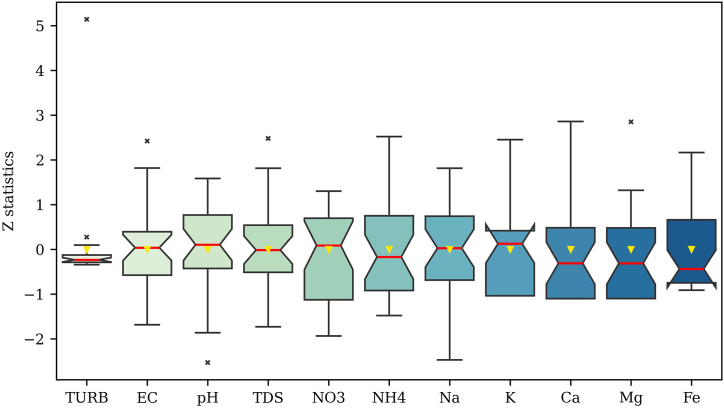
A statistical summary of the GW quality indicators in Bhola district.

In Fig. 3, a comprehensive statistical summary of GWQ indicators is presented. The majority of these indicators adhere to the permissible limits outlined by the Environmental Conservation Rules (ECR), Bangladesh, specifically designed for drinking water (refer to Table S2), except for K, Ca, and Mg (Fig. 3). The figure showcases the broad range of TURB, averaging 2.08 NTU. The EC demonstrated moderate variability, with an average of 578.21 μS/cm, while pH maintains a near-neutral average of 7.78. The TDS exhibited variability within the range of 130–510 mg/L. Both NO_3_^−^ and NH_4_^+^ concentrations displayed moderate variability around mean values of 5.39 mg/L and 0.91 mg/L, respectively. Concentration of Na shows variability around an average of 59.64 mg/L, and K averages of 9.46 mg/L with moderate variability. The level of Ca and Mg varied with an average of 70.43 mg/L and 40.50 mg/L, respectively. Concentration of Fe displayed variability around an average of 0.33 mg/L. Notably, the exceedance of certain indicators beyond established limits may be attributed to factors such as geological characteristics, anthropogenic activities, or natural variations in GW composition. Furthermore, the research employed Pearson's correlation technique to determine the interrelationships among WQ indicators across different sampling sites [87]. Fig. S2 illustrates Pearson's correlation results of various WQ indicators. As shown in Fig. S2, a significant positive association between EC and TDS is observed, consistent with the authors' previous study on GW resources [88].

### 3.2 Results of the RMS-WQI model

**Fig. 4:**
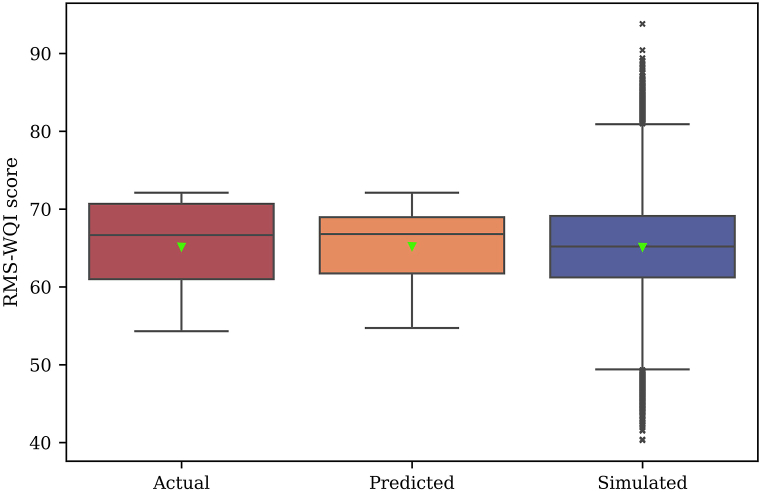
Comparison between actual and predicted RMS-WQI scores.

To maintain their designation of “good” WQ, all states must implement effective water resource management practices. The WQI model has become a widely employed tool for evaluating WQ due to its straightforward application and result interpretation. In this study, the RMS-WQI model, utilizing an unweighted method, was applied to assess the GWQ in the Bhola district. The statistical summary of the RMS-WQI model, depicted in Fig. 4, reveals WQI values ranging from 54.3 to 72.1, with an average WQI of 65.2; whereas Fig. S3 presents point-wise RMS-WQI across various sampling sites.

Fig. S4 depicts the spatial distribution of RMS-WQI scores, showcasing both actual and predicted values across sampling sites. The analysis indicates notably higher WQI scores in Bhola Sadar compared to Char Fashion, primarily attributed to breaches in guideline limits for elements such as K, Ca, and Mg in the majority of sampling sites within Char Fashion (See Table S3). The EBK interpolated scores of the RMS-WQI further validate the heightened scores observed in Bhola Sadar (Fig. S5). The assessment of geographical variation underscores the proficiency of RMS-WQI models in evaluating GW status, given their spatial resolution capabilities. Fig. S5 also reveals that there is no significant variation in WQI scores concerning the spatial resolution of acquirers in the study area. Consequently, the results of spatial variation suggest that the RMS-WQI model could be an effective tool for assessing GWQ across diverse geographical extents.

### 3.3 Prediction results of RMS-WQI model

#### 3.3.1 ML model evaluation results

**Fig. 5:**
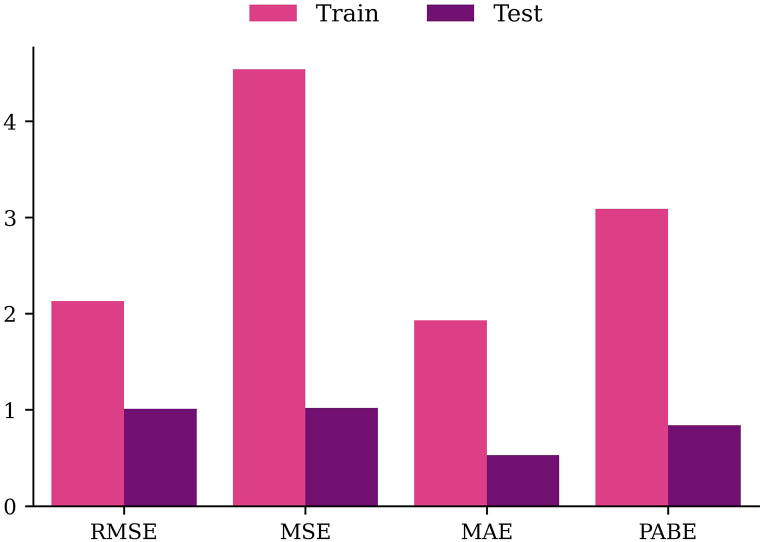
Model performance matrix during training and testing phase.

The evaluation of XGBoost model performance in predicting RMS-WQI scores incorporated four key metrics: RMSE, MSE, MAE, and PABE, as illustrated in Fig. 5. A comparison between the training and testing metrics provides insights into the model's ability to generalize. Ideally, the lower values in testing metrics (RMSE: 1.01, MSE: 1.02, MAE: 0.53, PABE: 0.84) compared to the training metrics (RMSE: 2.13, MSE: 4.54, MAE: 1.93, PABE: 3.09) indicate effective learning and accurate prediction of RMS-WQI scores on new, unseen data. In this context, the XGBoost model demonstrated promising performance, exhibiting precision and reliability in predicting WQ. These findings align with recent literature on XGBoost model performance. The relatively low testing error values underscore the model's potential as a valuable tool for predicting RMS-WQI scores in real-world scenarios.

#### 3.3.2 Effects of hyper-parameters on the prediction capabilities

For extracting reliable and robust predicting performance from any ML model(s), hyperparameters play a crucial role by controlling the learning process. In terms of WQ prediction, hyperparameters have tremendous impact on the accuracy and precision of predictive models. Table 2 provides the optimized hyperparameters and their optimal values for the XGB-OPT model, offering the best combination for predicting GW quality.

**Table 2:** Optimized hyperparameters and their optimal values for XGB model.

Model attributes	Optimized value
booster	gbtree
lambda	0.081
alpha	1.631
max_depth	11
n_estimators	230
learning_rate	0.034
subsample	0.939
colsample_bytree	0.903
min_child_weight	5
Optimizer	Optuna

The choice of an optimal lambda value (0.081) and alpha value (1.631) ensures a delicate balance, allowing the model to deal with overfitting issues through the addition of penalty term to the loss function. The map_depth function determines the maximum depth of trees in the model where larger and smaller values can lead to overfitting and underfitting issues, respectively. The max_depth of 11 balances the above-mentioned issues and offered a superior predictive performance. Additionally, the meticulous selection of n_components (230) in dimensionality reduction focuses on optimizing model's accuracy by providing careful attention on the crucial WQ aspects. The learning rate (0.034) controls the step size to which optimizer can update to the weights. Moreover, the subsample value (0.939) and colsample_bytree value (0.903) aided the model to prevent overfitting issue. The integration of the OPT optimizer further enhances the performance of the XGBoost algorithm for predicting GWQ.

#### 3.3.3 Comparison between actual and predicted WQI score

The statistical summary of actual, predicted, and simulated RMS-WQI scores for the GW in the study domain is presented in Fig. 4. Additionally, Fig. S6 offers a point-wise comparison of actual (computed) and predicted RMS-WQI scores at each sampling site. Fig. 4 illustrates minimal discrepancies between actual and predicted WQI scores, with the exception of simulation scores. No outliers were identified in the predicted RMS-WQI scores, aligning with the absence of outliers in the actual RMS-WQI scores (Fig. 4). Upon point-wise comparison, most sampling sites showed no significant differences between actual and predicted WQI scores, except for sites S1, S9, S10, S13, S19, S22, and S26 (Fig. S6). To further validate these differences, the research employed Tukey's HSD pair-wise comparison analysis following the methodology of Ref. [33]. The results of Tukey's HSD analysis revealed no statistically significant differences between actual and predicted scores at p < 0.01 with a 99 % confidence level, although slight differences were observed for the RMS-WQI scores at seven sampling sites (Fig. S7).

### 3.4 Model sensitivity results

**Fig. 6:**
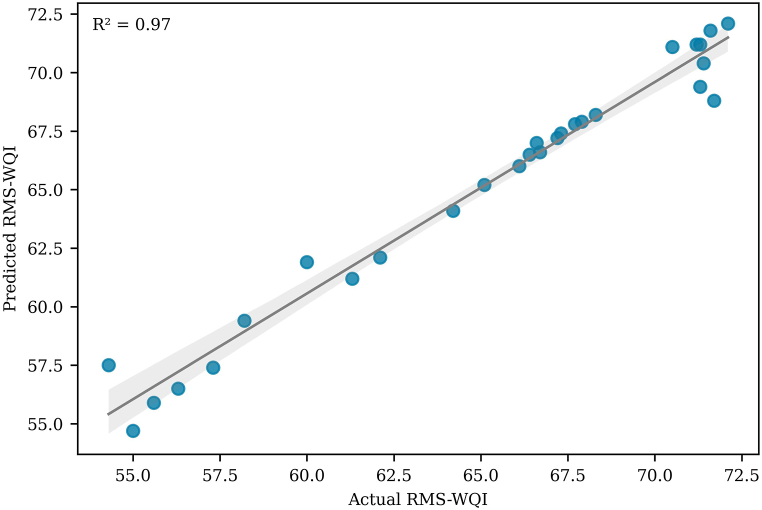
RMS-WQI model's sensitivity assessment for predicting WQI scores.

The coefficient of determination (R^2^) serves as a fundamental tool in analyzing the sensitivity of a model and is frequently employed in the literature for this purpose. This method has found application in recent studies evaluating model sensitivity in water research [[89], [90], [91]]. The sensitivity of the RMS-WQI model over the research period is depicted in Fig. 6. The unweighted RMS-WQI models demonstrate a high level of agreement (R^2^ = 0.97) between model input and output, as illustrated in Fig. 6. This consistency in sensitivity results aligns with previous investigations by Refs. [26,35,39,41,47]. Consequently, it can be asserted that the model exhibits outstanding performance in terms of sensitivity for evaluating GWQ, as evidenced by the robust R^2^ findings.

**Fig. 7:**
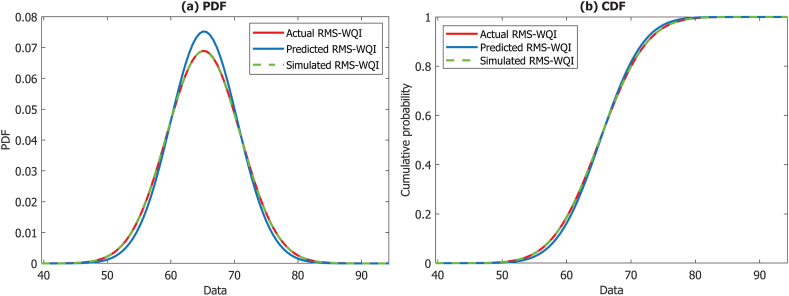
Comparison of the PDF and CDF plots (actual, predicted and simulated) for the RMS-WQI models over the study period.

### 3.5 Model uncertainty results

In evaluating uncertainty within the WQI model, this study employed two statistical measures: PDF and CDF, following the methodologies outlined by Ref. [32]. Detailed information on the methodology can be found in Ref. [32]. Fig. 7 illustrates the PDFs and CDFs results of the RMS-WQI scores (actual, predicted, and simulated) in the Bhola district throughout the study period. Analysis of these figures indicates no significant variation between actual and simulated WQI scores in both PDFs and CDFs. However, minimal variations in the PDFs and CDFs were observed for predicted WQI values (Fig. 7). Notably, the unweighted RMS-WQI model, when scrutinized for model uncertainty, demonstrates promising potential in assessing GWQ of the Bhola district. The PDFs and CDFs results also suggest that the RMS-WQI could be adeptly applied for handling large datasets, such as climatic and simulation data, with increased accuracy in terms of model uncertainty. These findings align with the outcomes of the authors' earlier investigations [29,32,36,[39], [40], [41]], which also delved into the model's uncertainty in evaluating WQ.

### 3.6 Efficiency of the RMS-WQI model for assessing GW quality

In assessing model efficiency, this study employed the methodology proposed by Ref. [39]. The selection of this methodology is grounded in its recent widespread adoption in various water research studies, reflecting its relevance and applicability in the field. Fig. S8 illustrates the model efficiency results, encompassing NSE and MEF. The NSE index scenarios, denoted as S1 to S28, predominantly function as indicators of model performance. Generally, values close to 1 across these scenarios signify a high level of agreement between observed and simulated values, reflecting successful model performance. However, the presence of a notable outlier in sampling site S17 with a value of −1.17, raises concerns about potential inaccuracies in the model for that specific scenario. On the other hand, MEF scenarios, ranging from 0 to 1, offer insights into the efficiency of the model in representing observed data In terms of MEF, values close to 0 in majority sampling sites implied favourable model performance, indicating potential agreement between predicted and observed data (Fig. S8).However, a higher MEF value was found in S17 where the model may face challenges in accurately representing observed data. The NSE and MEF results collectively suggest that the RMS-WQI approaches could be effective for evaluating and predicting GWQ. Furthermore, these findings align with recent studies in the literature, reinforcing the validity of the model efficiency outcomes.

### 3.7 Model eclipsing and ambiguity results

This study employed the methodologies outlined by Ref. [41] to evaluate the performance of the RMS-WQI model in addressing issues related to model eclipsing and ambiguity. The results of this analysis are summarized in Table S4. The statistics revealed that ambiguity issues were identified at three sites (11 %), and eclipsing problems were observed at 11 monitoring sites. While eclipsing problems affected 39 % of the sample locations, the WQ condition, as categorized in Table 1, did not undergo significant changes due to these issues. The analysis of eclipsing and ambiguity indicated that the RMS-WQI model remains a useful and practical approach for calculating WQI scores.

## 4 Assessment of GWQ in the Bhola district

**Fig. 8:**
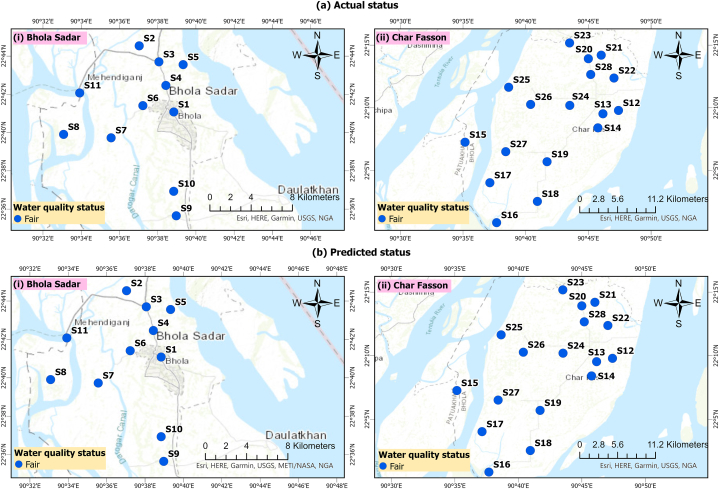
Spatial variation of WQ quality status across various sampling sites in the study domain.

The primary aim of the WQI model is to evaluate WQ status using a specified classification scheme. In this study, with the objective of assessing GWQ in the study area, the classification scheme proposed by Ref. [38] (outlined in Table 1) was employed. Fig. 8 illustrates the spatial variation in GWQ status across the study domain using the RMS-WQI model. The WQ at all sites consistently fell within the “fair” category. Moreover, the predicted scores from the RMS-WQI model align with this classification, highlighting the model's effectiveness in assessing GWQ by synthesizing information from various WQ indicators (Fig. 8b). The “fair” classification for GWQ implies potential health concerns for long-term usage (Table 1), emphasizing the need for purification before consumption and other health-related applications as outlined by Ref. [38].

It is crucial to acknowledge that several studies have documented the degradation of GWQ, especially in the coastal areas of Bangladesh, including the Bhola district [4,55,67]. Factors contributing to this degradation include excessive abstraction, improper disposal of industrial and municipal wastes, intensified application of agrochemicals, land use changes, and saline water intrusion [4,[92], [93], [94]]. Therefore, the present findings on GW resources of Bhola district align with observations from prior studies, underscoring the consistent challenges faced by the region. Furthermore, the present research work demonstrated the potential utilization of RMS-WQI model, which could be a reliable tool for assessing coastal GWQ by researchers and local authorities. It is crucial to point out that the inadequacy to expand the temporal resolution over the current study domain constitutes the only limitation for this study. However, the application of RMS-WQI model in assessing coastal GWQ considering seasonal GW database manifests the scope for further research works.

## 5 Study implications on SDGs

In this era of sustainable development approach, it is crucial to highlight the significance of the data-driven models for achieving SDGs particularly for water resources management [95,96]. The SDGs are the global tools for achieving a sustainable future for humanity and covers a range of economic, environmental, and social attributes. Details of the SDGs can be found in Ref. [97]. In terms of this current research, the utilization of RMS-WQI model in rating coastal GWQ would provide reliable information on GW state that would ultimately assist in achieving SDG no. 6 (clean water and sanitation). Moreover, the accurate and reliable outcome of the RMS-WQI model in rating coastal GW resources would further extend its potentials in achieving SDG 2 (zero hunger; under the SDG target 2.4-sustainable food production systems and resilient agricultural practices), SDG 3 (good health and well-being; under the SDG target 3.1- reduce the global maternal mortality rate and target 3.9-reduce illnesses and death from hazardous chemicals and pollution), SDG 12 (responsible consumption and production; under the SDG target 12.2 - sustainable management of natural resources), SDG 13 (contributes to climate change adaptation strategies by providing a reliable alternative resource less impacted by pollution and environmental stressors than surface water), SDG 15 (life on land; under the SDG target 15.1-on the conservation of freshwater ecosystems and their services). Furthermore, GW state information from the RMS-WQI model would have indirect implications on GW discharge to coastal areas for marine ecosystem (SDG 14) while access to safe GW resources provides opportunities in protecting peace (SDG 16).

## 6 Conclusion

The goal of this study was to assess GW quality in the Bhola district, close to the Bay of Bengal, using an advanced WQ model using an advanced WQ model incorporating ML approaches. A thorough analysis was conducted to evaluate the model's efficacy using a variety of statistical metrics, including those for prediction performance (RMSE, MSE, MAE, and PABE), model sensitivity (R^2^), model efficiency (NSE and MEF), and consideration of uncertainty (eclipsing and ambiguity). The research's conclusions may be summed up as follows. First off, the majority of WQ indicators in our study region were determined to be within guideline levels across several sample locations, with the exception of K, Ca, and Mg, whose threshold value exceeded the allowed limit. Second, the unweighted RMS-WQI model consistently assigned “fair” categories to WQ. The performance evaluation also showed that the RMS-WQI model with the XGBoost algorithm was effective in accurately assessing GWQ , with all performance indicators (RMSE, MSE, MAE and PABE) demonstrating their reliability in reducing model uncertainty (less than 1 %) without running into significant eclipsing or ambiguity problems. A good degree of agreement (R^2^ = 0.97) between the model input and output was found through the sensitivity analysis, demonstrating the models' sensitivity to the spatial resolution of water bodies. Additionally, the NSE and MEF findings demonstrated the effectiveness of the WQI model in producing precise WQI values. In order to lessen the uncertainty in the WQI model, the results of this study would have also been considerably more beneficial in precisely forecasting WQI scores at each monitoring site. One of the limitations of this study is the inability to sufficiently evaluate the WQ in terms of temporal resolution. Therefore, future research should consider the seasonal dynamics for assessing the GWQ of the study region. Although the current study is based on single season data, the findings suggested that the RMS-WQI could be a reliable tool for rating GW of coastal regions and finally informing relevant stakeholders and policymakers for implementing adequate monitoring strategies.

## Data availability statement

No data was used for the research described in the article.

## CRediT authorship contribution statement

**Md Galal Uddin:** Writing – review & editing, Writing – original draft, Visualization, Validation, Supervision, Software, Resources, Project administration, Methodology, Investigation, Funding acquisition, Formal analysis, Data curation, Conceptualization. **M.M. Shah Porun Rana:** Writing – review & editing, Writing – original draft, Visualization, Software, Data curation. **Mir Talas Mahammad Diganta:** Writing – review & editing, Writing – original draft, Visualization, Validation, Software, Methodology, Investigation, Formal analysis, Data curation, Conceptualization. **Apoorva Bamal:** Writing – review & editing. **Abdul Majed Sajib:** Writing – review & editing. **Mohamed Abioui:** Writing – review & editing. **Molla Rahman Shaibur:** Writing – review & editing, Investigation, Formal analysis. **S.M. Ashekuzzaman:** Writing – review & editing. **Mohammad Reza Nikoo:** Writing – review & editing. **Azizur Rahman:** Writing – review & editing, Validation, Methodology, Data curation. **Md Moniruzzaman:** Writing – review & editing. **Agnieszka I. Olbert:** Writing – review & editing, Writing – original draft, Supervision, Resources, Formal analysis.

## Declaration of competing interest

The authors declare that they have no known competing financial interests or personal relationships that could have appeared to influence the work reported in this paper.
