# Supplementary material for: Enhancing groundwater quality assessment in coastal area: A hybrid modeling approach
Source: Heliyon. 2024 Jun 19;10(13):e33082. doi: 10.1016/j.heliyon.2024.e33082 (PMC11255574; doi:10.1016/j.heliyon.2024.e33082)
Supplement: Multimedia component 1 [file mmc1.docx]

**Supplementary materials**

**Enhancing Groundwater Quality Assessment in Coastal Area: A Hybrid Modeling Approach**

**Md Galal Uddin^a,b,c,d,*^, M. M. Shah Porun Rana^e^, Mir Talas Mahammad Diganta^a,b,c,d^, Apoorva Bamal^a,b,c,d^, Abdul Majed Sajib^a,b,c,d^, Mohamed Abioui^f,g,h^, Molla Rahman Shaibur^i^, S. M. Ashekuzzaman^j^, Mohammad Reza Nikoo^k^, Azizur Rahman^l,m^, Md Moniruzzaman^e^, Agnieszka I. Olbert^a,b,c,d^**

^a^ School of Engineering, University of Galway, Ireland

^b^ Ryan Institute, University of Galway, Ireland

^c^ MaREI Research Centre, University of Galway, Ireland

^d^ Eco-HydroInformatics Research Group (EHIRG), Civil Engineering, University of Galway, Ireland

^e^ The Department of Geography and Environment, Jagannath University, Dhaka, Bangladesh

^f^ Geosciences, Environment and Geomatics Laboratory (GEG), Department of Earth Sciences, Faculty of Sciences, Ibnou Zohr University, Agadir, Morocco

^g^ MARE-Marine and Environmental Sciences Centre-Sedimentary Geology Group, Department of Earth Sciences, Faculty of Sciences and Technology, University of Coimbra, Coimbra, Portugal

^h^ Laboratory for Sustainable Innovation and Applied Research, Universiapolis—International University of Agadir, Agadir, Morocco

^i^ Laboratory of Environmental Chemistry, Department of Environmental Science and Technology, Faculty of Applied Science and Technology, Jashore University of Science and Technology, Jashore 7408, Bangladesh

^j^ Department of Civil, Structural and Environmental Engineering, and Sustainable Infrastructure Research & Innovation Group, Munster Technological University, Cork, Ireland

^k^ Department of Civil and Architectural Engineering, Sultan Qaboos University, Muscat, Oman

^l^ School of Computing, Mathematics and Engineering, Charles Sturt University, Wagga Wagga, Australia

^m^ The Gulbali Institute of Agriculture, Water and Environment, Charles Sturt University, Wagga Wagga, Australia

***Corresponding author:** Md Galal Uddin, Postdoctoral Researcher, Civil Engineering, College of Science and Engineering, University of Galway, Ireland. Email: [mdgalal.uddin@universityofgalway.ie](mailto:mdgalal.uddin@universityofgalway.ie)

**Table S1.** Sampling sites within the study domain.

| Area | Sampling site | Latitude | Longitude |
| --- | --- | --- | --- |
| Bhola Sadar upazila | S1 | 90.64735833 | 22.68467222 |
|  | S2 | 90.61721667 | 22.74228333 |
|  | S3 | 90.63439167 | 22.72828611 |
|  | S4 | 90.6405 | 22.70774444 |
|  | S5 | 90.65555278 | 22.72592222 |
|  | S6 | 90.62040833 | 22.69022222 |
|  | S7 | 90.59268333 | 22.66213611 |
|  | S8 | 90.55132222 | 22.66512222 |
|  | S9 | 90.64955833 | 22.59415833 |
|  | S10 | 90.64733889 | 22.61553611 |
|  | S11 | 90.56534444 | 22.70125278 |
| Char Fasson upazila | S12 | 90.79013333 | 22.16323333 |
|  | S13 | 90.76940833 | 22.15882222 |
|  | S14 | 90.762725 | 22.14017222 |
|  | S15 | 90.586 | 22.121 |
|  | S16 | 90.62812222 | 22.01412222 |
|  | S17 | 90.61914167 | 22.06710278 |
|  | S18 | 90.68232222 | 22.04232222 |
|  | S19 | 90.69513333 | 22.09502222 |
|  | S20 | 90.75 | 22.23199722 |
|  | S21 | 90.76709722 | 22.23667778 |
|  | S22 | 90.78413333 | 22.20623333 |
|  | S23 | 90.725 | 22.253 |
|  | S24 | 90.725275 | 22.16993333 |
|  | S25 | 90.6441 | 22.19405278 |
|  | S26 | 90.67322778 | 22.171175 |
|  | S27 | 90.64016944 | 22.10851111 |
|  | S28 | 90.75313333 | 22.21104167 |


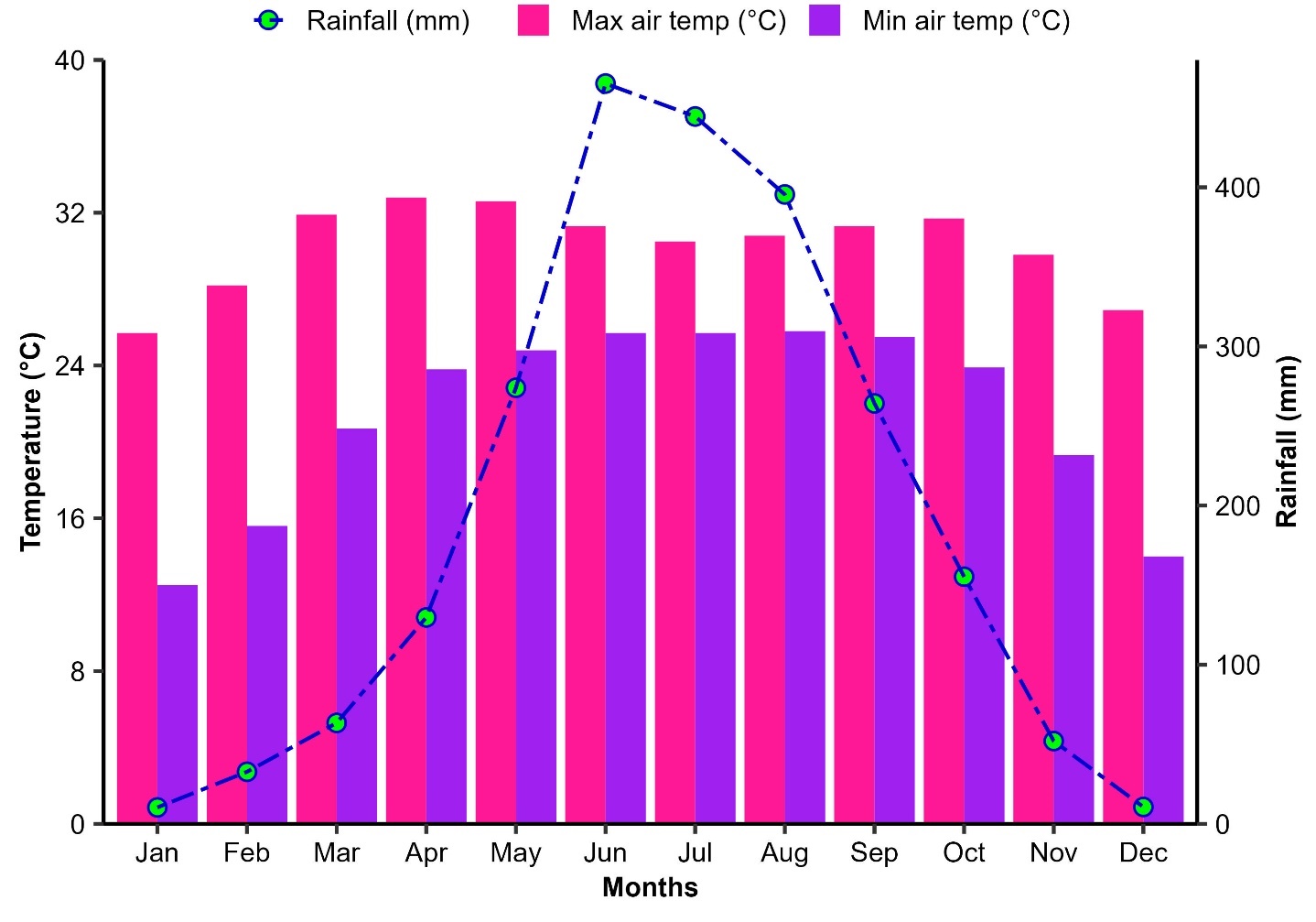


**Fig. S1.** Meteorological attributes of the study domain (data obtained from Bangladesh Meteorological Department; <https://live4.bmd.gov.bd/>).

**Table S2.** GW quality indicators and guidelines limits (ECR, 2023).

| WQ indicators | Abbreviation | Unit | Guideline limits |
| --- | --- | --- | --- |
| Turbidity | TURB | NTU | 5 |
| Electrical conductivity | EC |  | 1500^*^ |
| pH | - | - | 6.5-8.5 |
| Total dissolved solids | TDS | mg/L | 1000 |
| Nitrate | NO_3_^‑^ | mg/L | 45 |
| Ammonium | NH_4_^+^ | mg/L | 1.5 |
| Sodium | Na | mg/L | 200 |
| Potassium | K | mg/L | 12 |
| Calcium | Ca | mg/L | 75 |
| Magnesium | Mg | mg/L | 30-35 |
| Iron | Fe | mg/L | 0.3-1 |
| ^*^ WHO (2017) | | | |

**Table S3.** Concentrations and sub-index values of each water quality indicator in 28 sampling sites. The guideline values (thresholds) adopted for the sampling sites are included at the bottom.

| Sampling Sites | Concentrations | | | | | | | | | | | Sub-index values | | | | | | | | | | | RMS score | Rank |
| --- | --- | --- | --- | --- | --- | --- | --- | --- | --- | --- | --- | --- | --- | --- | --- | --- | --- | --- | --- | --- | --- | --- | --- | --- |
|  | TURB (NTU) | EC (µS/cm) | pH | TDS (mg/L) | NO3 (mg/L) | NH4 (mg/L) | Na (mg/L) | K (mg/L) | Ca (mg/L) | Mg (mg/L) | Fe (mg/L) | TURB | EC | pH | TDS | NO3 | NH4 | Na | K | Ca | Mg | Fe |  |  |
| S1 | 0.47 | 570 | 7.97 | 270 | 7 | 0.42 | 65 | 5 | 80.16 | 46.06 | 0.16 | 90.6 | 62 | 100 | 73 | 84.4 | 72 | 67.5 | 58.3 | 0 | 0 | 84 | 70.5 | Fair |
| S2 | 0.5 | 650 | 8.03 | 330 | 7 | 0.46 | 65 | 10 | 48.09 | 27.66 | 0.2 | 90 | 56.6 | 100 | 67 | 84.4 | 69.3 | 67.5 | 16.6 | 35.8 | 20.9 | 80 | 67.9 | Fair |
| S3 | 1.47 | 620 | 7.21 | 300 | 7 | 0.35 | 60 | 10 | 64.13 | 36.85 | 0.68 | 70.6 | 58.6 | 35.5 | 70 | 84.4 | 76.6 | 70 | 16.6 | 14.4 | 0 | 32 | 55.6 | Fair |
| S4 | 0.78 | 510 | 7.8 | 250 | 6 | 0.32 | 55 | 5 | 96.19 | 55.27 | 0.2 | 84.4 | 66 | 100 | 75 | 86.6 | 78.6 | 72.5 | 58.3 | 0 | 0 | 80 | 71.3 | Fair |
| S5 | 1.04 | 590 | 8.06 | 290 | 5 | 0.17 | 65 | 5 | 64.13 | 36.85 | 0.09 | 79.2 | 60.6 | 100 | 71 | 88.8 | 88.6 | 67.5 | 58.3 | 14.4 | 0 | 91 | 72.1 | Fair |
| S6 | 0.64 | 630 | 7.81 | 350 | 6 | 0.55 | 75 | 15 | 48.09 | 27.66 | 0.9 | 87.2 | 58 | 100 | 65 | 86.6 | 63.3 | 62.5 | 0 | 35.8 | 20.9 | 10 | 62.1 | Fair |
| S7 | 0.44 | 510 | 7.73 | 250 | 4 | 0.68 | 45 | 5 | 96.19 | 55.27 | 0.18 | 91.2 | 66 | 100 | 75 | 91.1 | 54.6 | 77.5 | 58.3 | 0 | 0 | 82 | 71.2 | Fair |
| S8 | 1.32 | 700 | 7.7 | 360 | 7 | 0.42 | 65 | 10 | 48.09 | 27.66 | 0.36 | 73.6 | 53.3 | 100 | 64 | 84.4 | 72 | 67.5 | 16.6 | 35.8 | 20.9 | 64 | 64.2 | Fair |
| S9 | 0.45 | 640 | 7.71 | 310 | 4 | 0.36 | 55 | 10 | 48.09 | 27.66 | 0.06 | 91 | 57.3 | 100 | 69 | 91.1 | 76 | 72.5 | 16.6 | 35.8 | 20.9 | 94 | 71.7 | Fair |
| S10 | 0.85 | 910 | 7.83 | 430 | 7 | 1.46 | 85 | 10 | 80.16 | 46.06 | 1.07 | 83 | 39.3 | 100 | 57 | 84.4 | 2.66 | 57.5 | 16.6 | 0 | 0 | 0 | 54.3 | Fair |
| S11 | 1.49 | 900 | 7.64 | 450 | 5 | 1.12 | 80 | 15 | 64.13 | 36.85 | 0.03 | 70.2 | 40 | 100 | 55 | 88.8 | 25.3 | 60 | 0 | 14.4 | 0 | 97 | 61.3 | Fair |
| S12 | 2.52 | 270 | 8.12 | 130 | 6 | 0.78 | 50 | 10 | 64.13 | 36.85 | 0.07 | 49.6 | 82 | 100 | 87 | 86.6 | 48.0 | 75 | 16.6 | 14.4 | 0 | 93 | 68.3 | Fair |
| S13 | 1.77 | 490 | 7.85 | 250 | 4 | 0.77 | 25 | 5 | 96.19 | 55.85 | 0.01 | 64.6 | 67.3 | 100 | 75 | 91.1 | 48.6 | 87.5 | 58.3 | 0 | 0 | 99 | 71.3 | Fair |
| S14 | 0.38 | 300 | 7.36 | 130 | 5 | 1.06 | 45 | 10 | 80.16 | 46.06 | 0.03 | 92.4 | 80 | 43.0 | 87 | 88.8 | 29.3 | 77.5 | 16.6 | 0 | 0 | 97 | 66.6 | Fair |
| S15 | 3.49 | 1020 | 7.6 | 510 | 4 | 1.42 | 80 | 15 | 48.09 | 27.66 | 0.17 | 30.2 | 32 | 100 | 49 | 91.1 | 5.33 | 60 | 0 | 35.8 | 20.9 | 83 | 56.3 | Fair |
| S16 | 0.71 | 780 | 7.45 | 360 | 7 | 0.87 | 45 | 10 | 96.19 | 55.27 | 0.55 | 85.8 | 48 | 47.5 | 64 | 84.4 | 42.0 | 77.5 | 16.6 | 0 | 0 | 45 | 55.0 | Fair |
| S17 | 0.77 | 280 | 8 | 170 | 3 | 1.59 | 75 | 5 | 96.19 | 55.27 | 1.02 | 84.6 | 81.3 | 100 | 83 | 93.3 | 0 | 62.5 | 58.3 | 0 | 0 | 0 | 65.1 | Fair |
| S18 | 0.88 | 520 | 7.9 | 260 | 4 | 1.26 | 70 | 10 | 48.09 | 27.66 | 0.13 | 82.4 | 65.3 | 100 | 74 | 91.1 | 16.0 | 65 | 16.6 | 35.8 | 20.9 | 87 | 66.7 | Fair |
| S19 | 0.3 | 410 | 7.55 | 200 | 5 | 1.46 | 60 | 5 | 64.13 | 36.85 | 0.04 | 94 | 72.6 | 100 | 80 | 88.8 | 2.66 | 70 | 58.3 | 14.4 | 0 | 96 | 71.4 | Fair |
| S20 | 2.57 | 420 | 7.77 | 210 | 6 | 0.71 | 40 | 5 | 80.16 | 46.06 | 0.32 | 48.6 | 72 | 100 | 79 | 86.6 | 52.6 | 80 | 58.3 | 0 | 0 | 68 | 66.4 | Fair |
| S21 | 1.29 | 570 | 7.72 | 280 | 6 | 0.96 | 50 | 10 | 48.09 | 27.66 | 0.03 | 74.2 | 62 | 100 | 72 | 86.6 | 36.0 | 75 | 16.6 | 35.8 | 20.9 | 97 | 67.7 | Fair |
| S22 | 0.76 | 710 | 7.46 | 360 | 4 | 1.36 | 65 | 15 | 64.13 | 36.85 | 0.12 | 84.8 | 52.6 | 48.0 | 64 | 91.1 | 9.33 | 67.5 | 0 | 14.4 | 0 | 88 | 58.2 | Fair |
| S23 | 1.18 | 420 | 8.02 | 210 | 5 | 0.76 | 50 | 5 | 64.13 | 36.85 | 0.02 | 76.4 | 72 | 100 | 79 | 88.8 | 49.3 | 75 | 58.3 | 14.4 | 0 | 98 | 71.6 | Fair |
| S24 | 1.1 | 610 | 8.14 | 300 | 6 | 1.05 | 60 | 5 | 80.16 | 46.06 | 0.07 | 78 | 59.3 | 100 | 70 | 86.6 | 30.0 | 70 | 58.3 | 0 | 0 | 93 | 67.3 | Fair |
| S25 | 0.6 | 580 | 7.83 | 280 | 4 | 1 | 40 | 5 | 64.13 | 36.85 | 0.56 | 88 | 61.3 | 100 | 72 | 91.1 | 33.3 | 80 | 58.3 | 14.4 | 0 | 44 | 66.1 | Fair |
| S26 | 0.4 | 620 | 7.79 | 300 | 7 | 2.17 | 75 | 20 | 48.09 | 27.66 | 1.05 | 92 | 58.6 | 100 | 70 | 84.4 | 0 | 62.5 | 0 | 35.8 | 20.9 | 0 | 60.0 | Fair |
| S27 | 1.4 | 650 | 7.95 | 330 | 4 | 1.75 | 70 | 15 | 64.13 | 36.85 | 0.78 | 72 | 56.6 | 100 | 67 | 91.1 | 0 | 65 | 0 | 14.4 | 0 | 22 | 57.3 | Fair |
| S28 | 28.6 | 310 | 7.89 | 150 | 6 | 0.22 | 55 | 15 | 128.26 | 73.7 | 0.22 | 0 | 79.3 | 100 | 85 | 86.6 | 85.3 | 72.5 | 0 | 0 | 0 | 78 | 67.2 | Fair |
| ECR 2023 | 5 | 1500* | 6.5-8.5 | 1000 | 45 | 1.5 | 200 | 12 | 75 | 30-35 | 0.3-1 |  |  |  |  |  |  |  |  |  |  |  |  |  |
|  | Breached criteria | | |  | Ambiguity problem | | |  | Eclipsing problem | | |  |  |  |  |  |  |  |  |  |  |  |  |  |


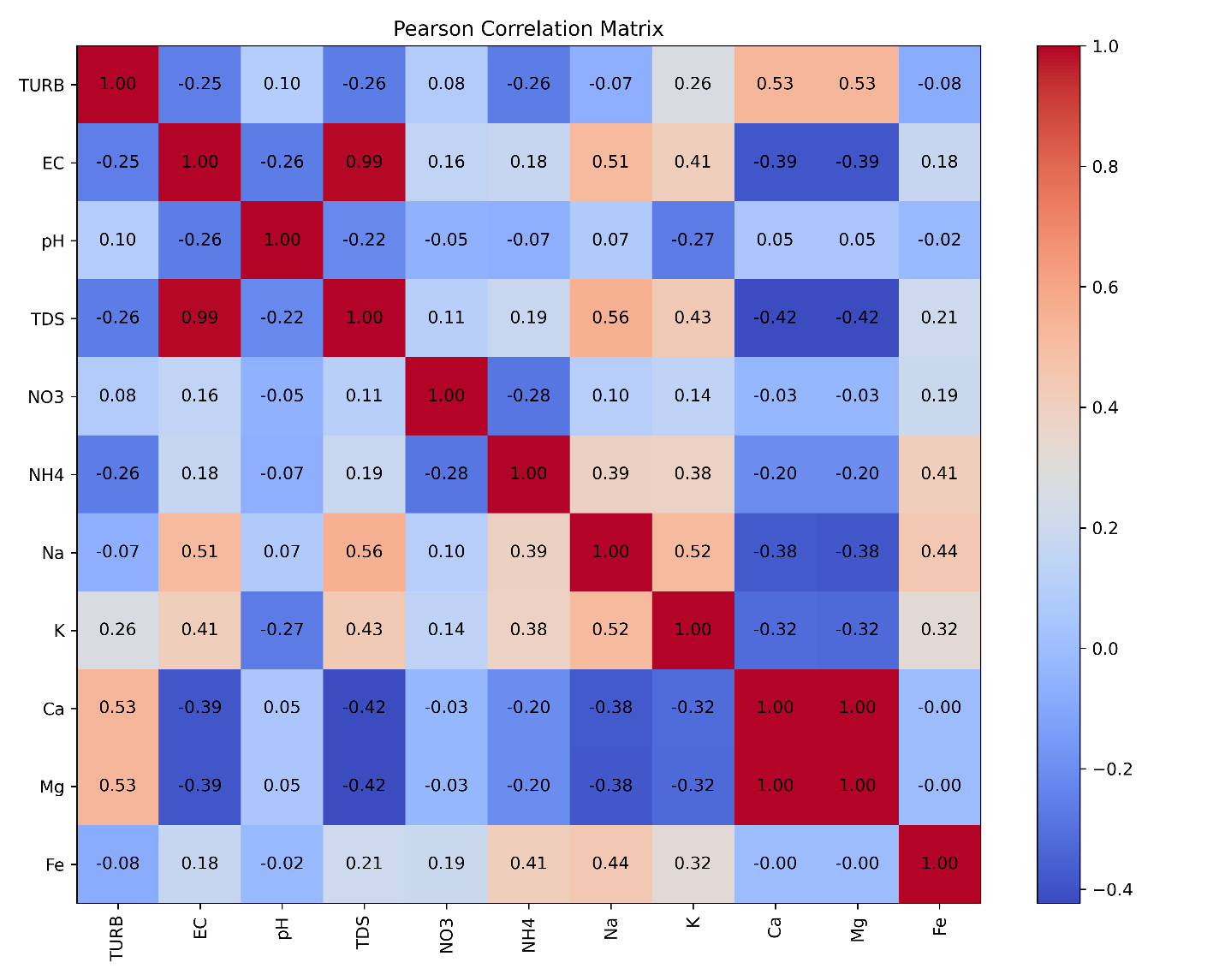


**Fig. S2.** Pearson’s correlation between GW quality indicators in Bhola district.


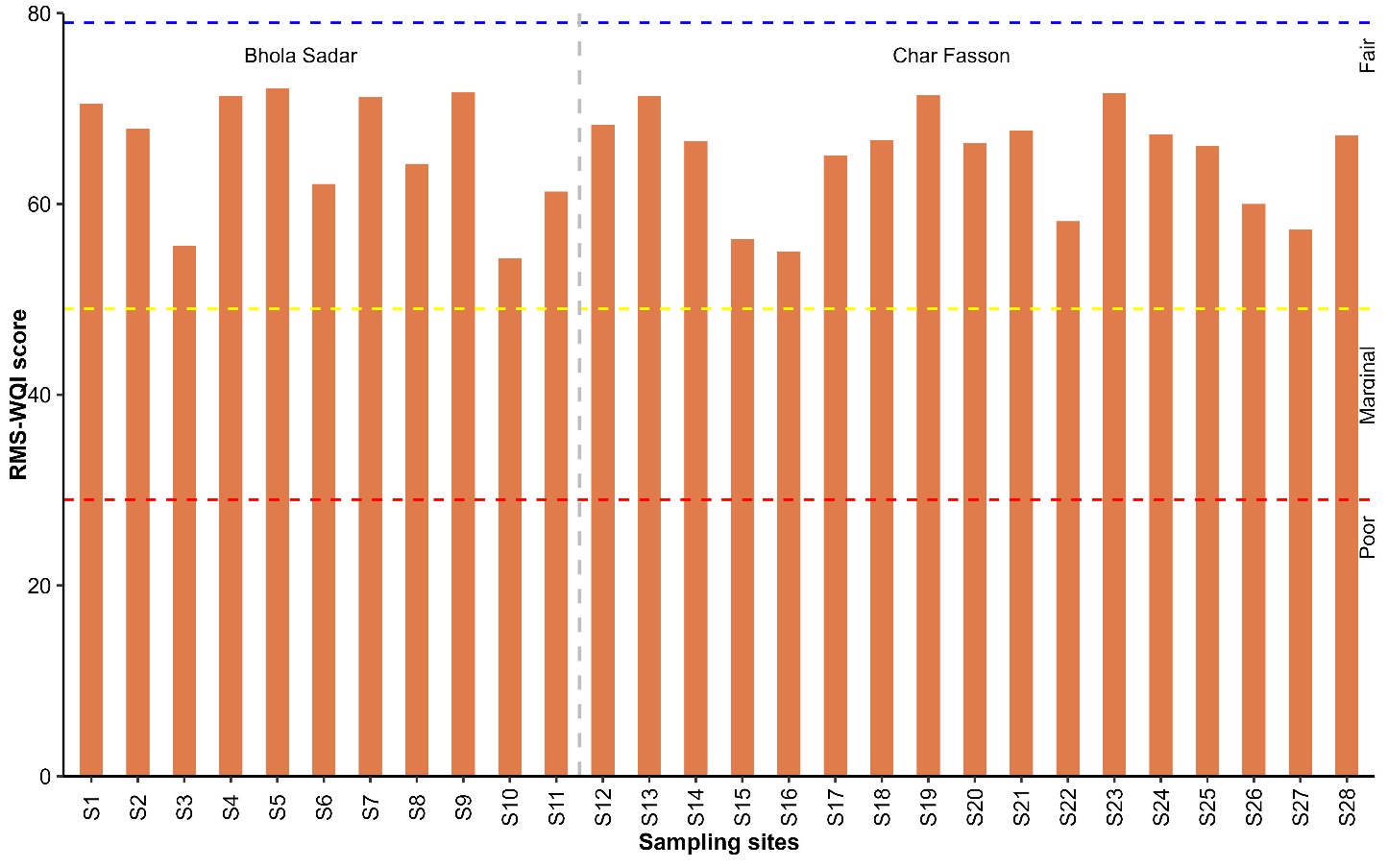


**Fig. S3.** Sampling site-based RMS-WQI score.


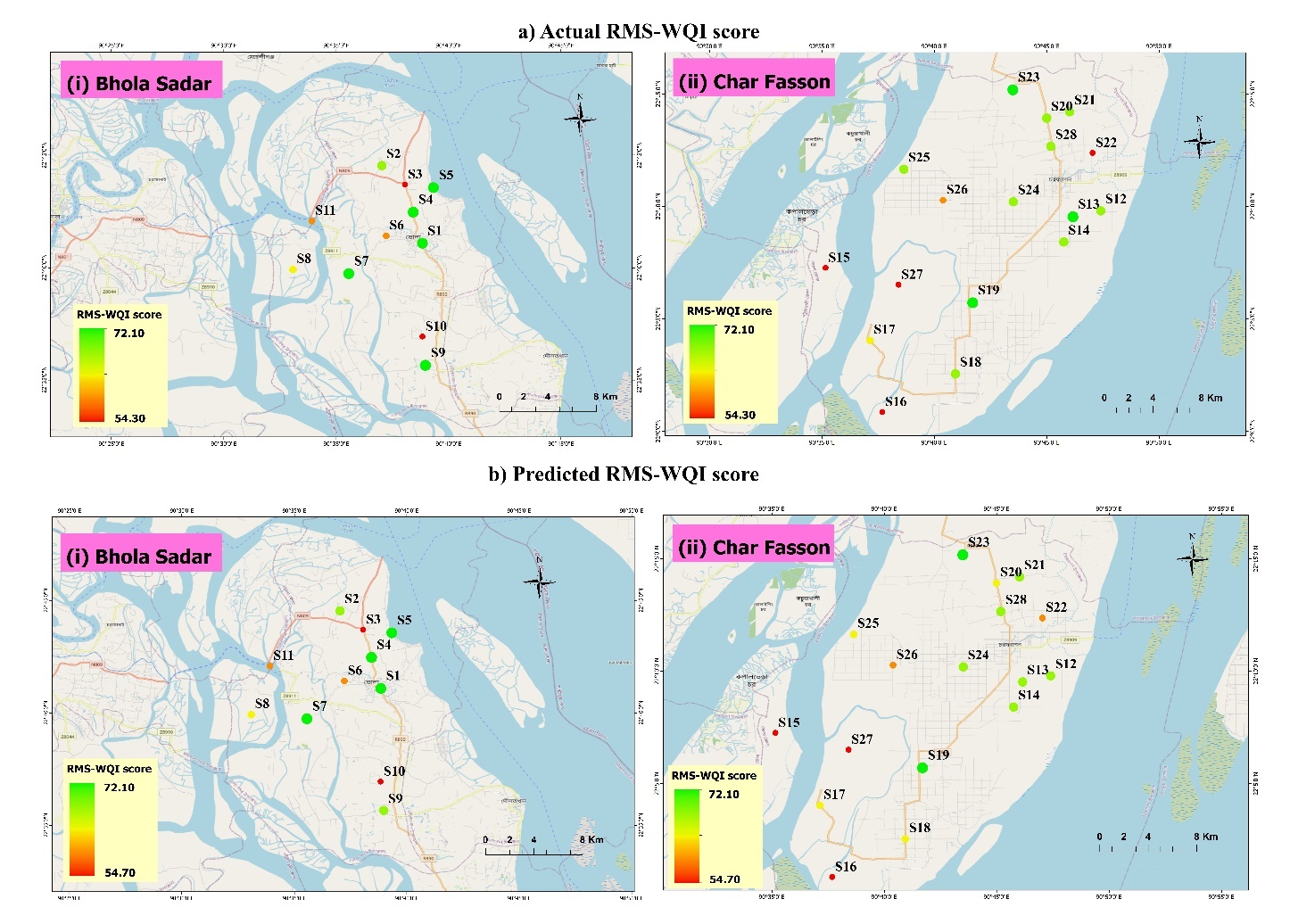


**Fig. S4.** Spatial variation of RMS-WQI score across the sampling sites in the study domain.


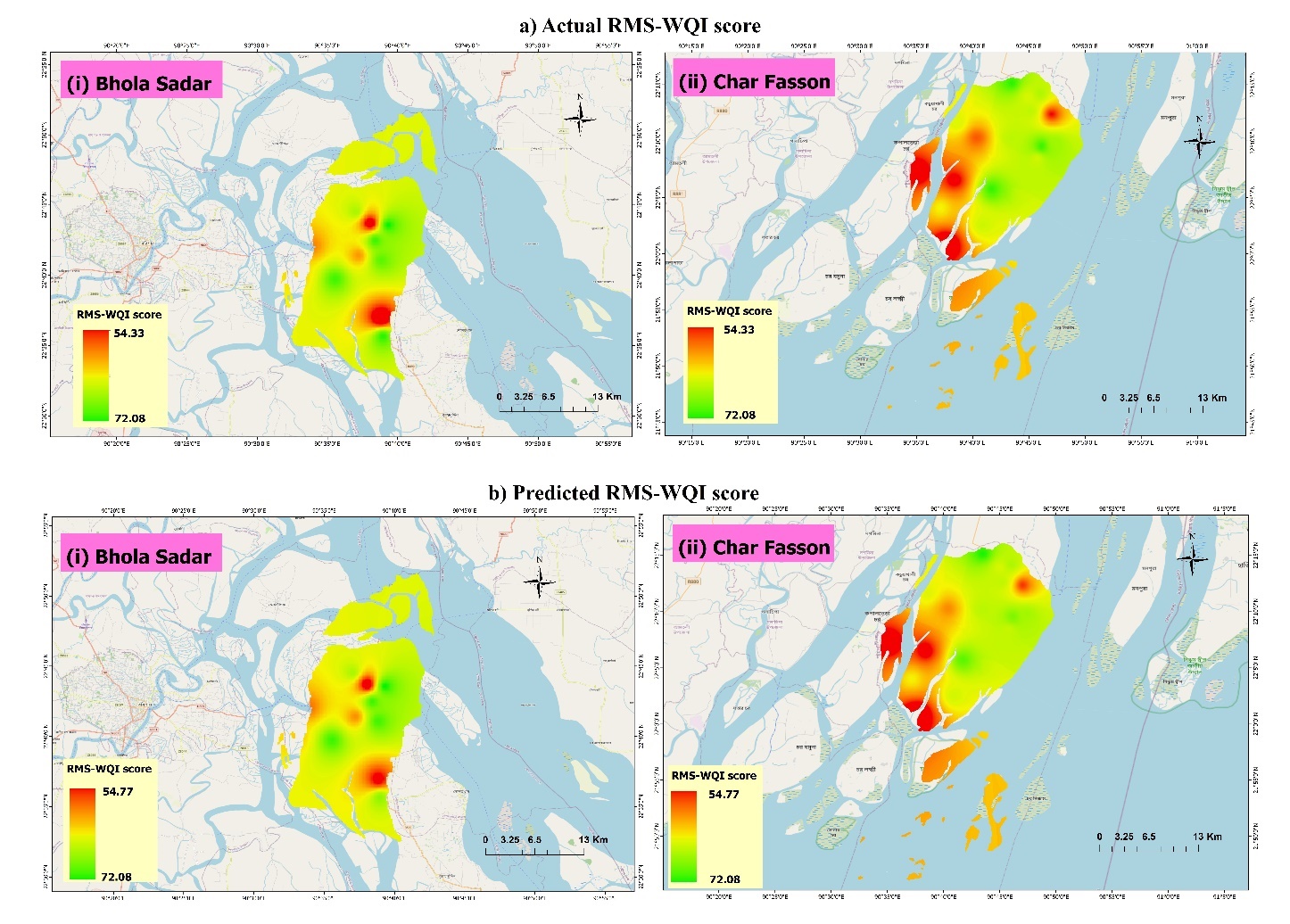


**Fig. S5.** Spatial variation of WQI score for actual and predicted model.


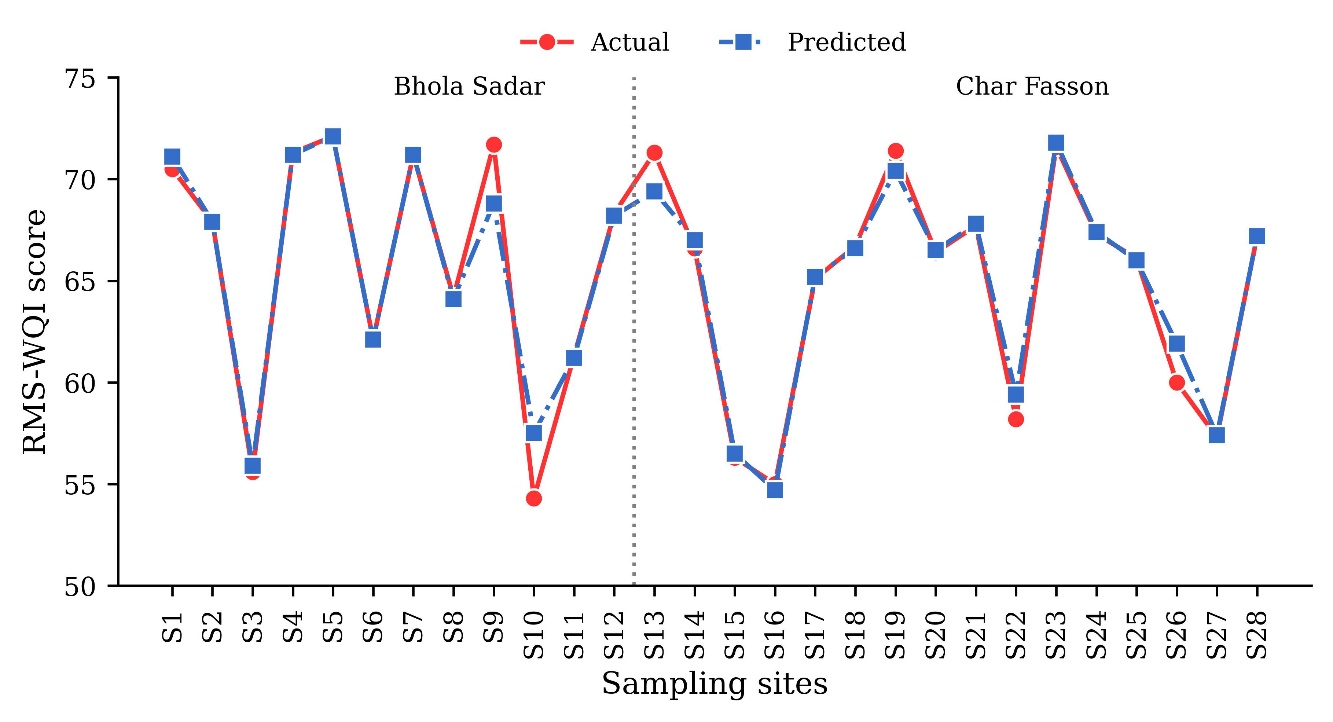


**Fig. S6.** Point-by-point RMS-WQI scores comparison across different sampling sites in the study domain.

**
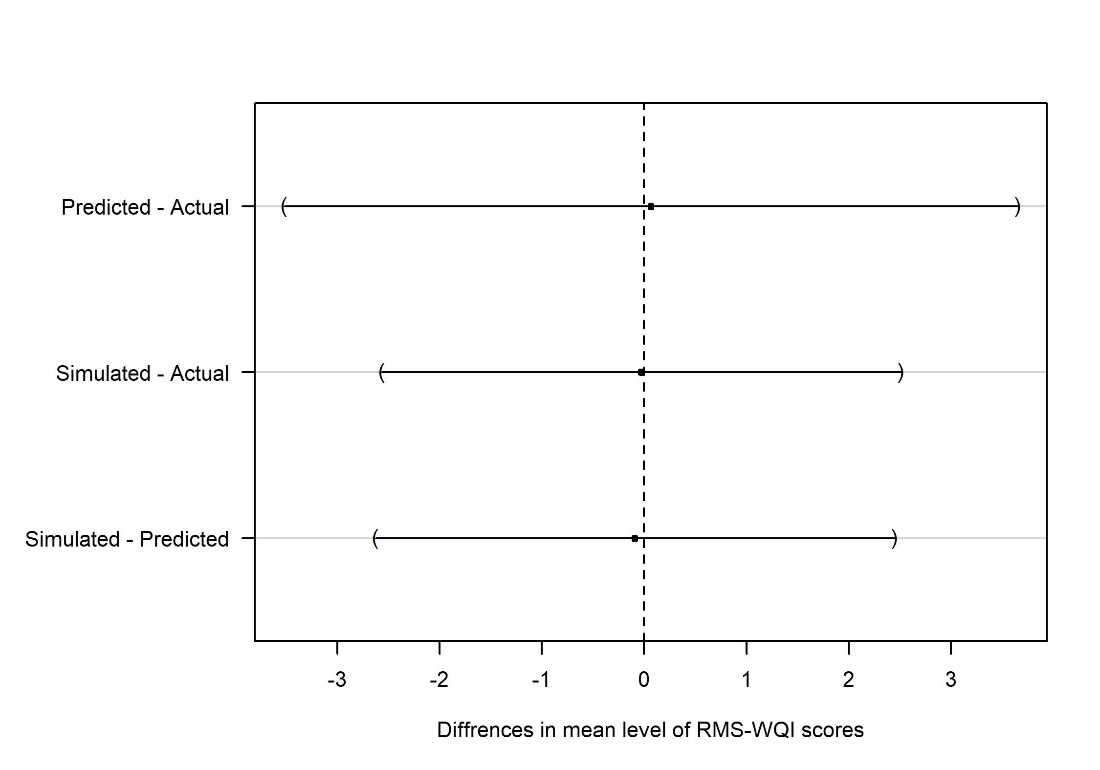
**

**Fig. S7.** Pair-wise comparison between the actual, predicted and simulated RMS-WQI scores with 95% CI from Tukey’s HSD.

***
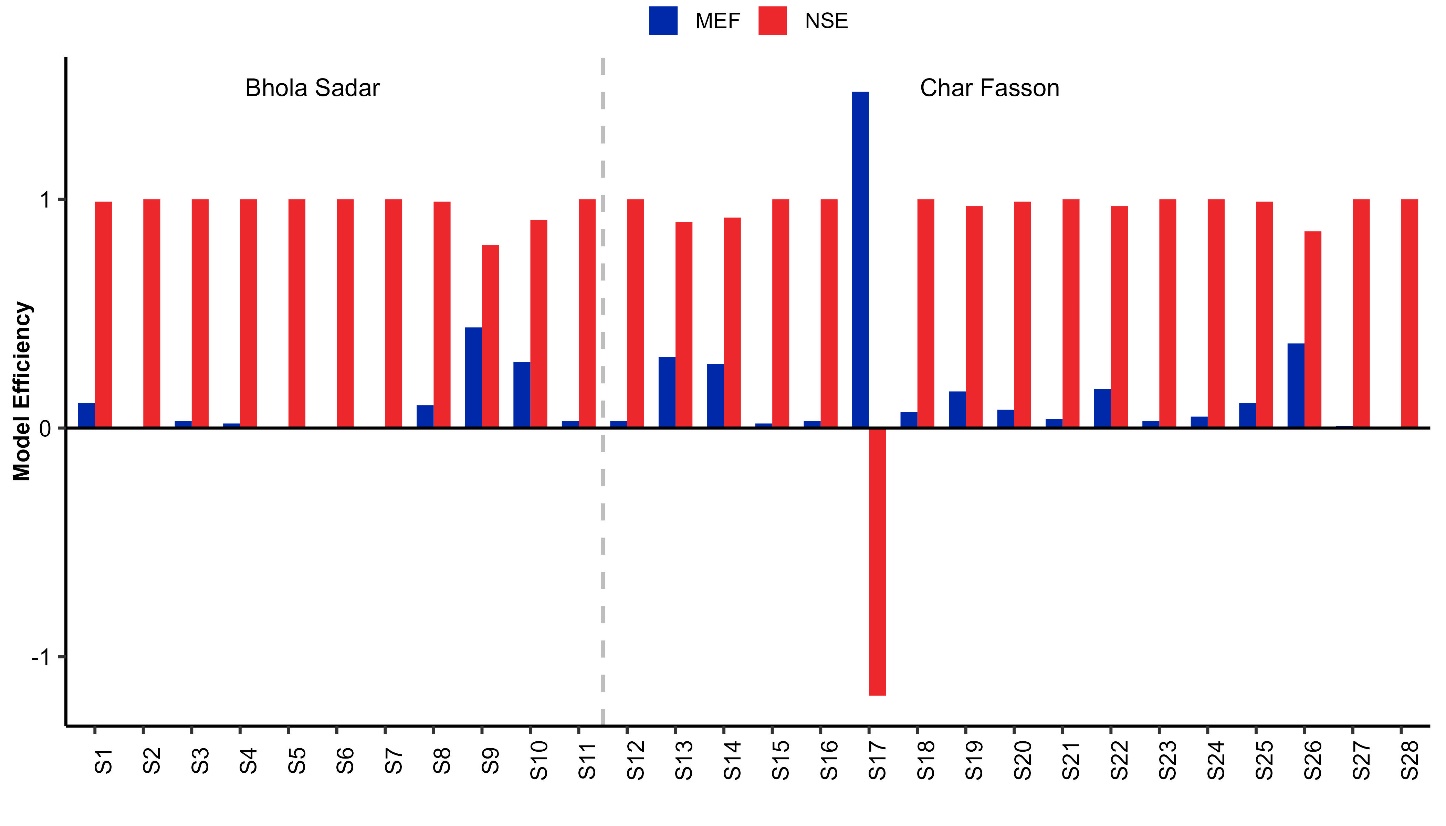
***

**Fig. S8.** The RMS-WQI model efficiency results across the samplings sites within the study domain.

**Table S4.** Model ambiguity and eclipsing results.

| Attributes | Statistics |
| --- | --- |
| Eclipsing | 11 (39%) |
| Ambiguity | 3 (11%) |
| Total sites | 28 |
